# Electrophysiological evaluation of extracellular spermine and alkaline pH on synaptic human GABA_A_ receptors

**DOI:** 10.1038/s41398-019-0551-1

**Published:** 2019-09-05

**Authors:** A. Limon, E. Delbruck, A. Yassine, D. Pandya, R. M. Myers, J. D. Barchas, F. Lee, Schatzberg, S. J. Watson, H. Akil, W. E. Bunney, M. P. Vawter, A. Sequeira

**Affiliations:** 1 Department of Psychiatry and Human Behavior. School of Medicine, University of California Irvine, Irvine, USA; 2 Department of Neurology, Mitchel Center for Neurodegenerative Diseases, School of Medicine, University of Texas Medical Branch at Galveston, Galveston, USA; 3 HudsonAlpha Institute for Biotechnology, Huntsville, AL USA; 4 Department of Psychiatry, Weill Cornell Medical College, New York, NY USA; 5 Department of Psychiatry & Behavioral Sciences, Stanford University, Palo Alto, CA USA; 6 Molecular and Behavioral Neurosciences Institute, University of Michigan, Ann Arbor, MI USA

**Keywords:** Physiology, Pharmacodynamics

## Abstract

Polyamines have fundamental roles in brain homeostasis as key modulators of cellular excitability. Several studies have suggested alterations in polyamine metabolism in stress related disorders, suicide, depression, and neurodegeneration, making the pharmacological modulation of polyamines a highly appealing therapeutic strategy. Polyamines are small aliphatic molecules that can modulate cationic channels involved in neuronal excitability. Previous indirect evidence has suggested that polyamines can modulate anionic GABA_A_ receptors (GABA_A_Rs), which mediate inhibitory signaling and provide a direct route to reduce hyperexcitability. Here, we attempted to characterize the effect that spermine, the polyamine with the strongest reported effect on GABA_A_Rs, has on human postmortem native GABA_A_Rs. We microtransplanted human synaptic membranes from the dorsolateral prefrontal cortex of four cases with no history of mental or neurological disorders, and directly recorded spermine effects on ionic GABA_A_Rs responses on microtransplanted oocytes. We show that in human synapses, inhibition of GABA_A_Rs by spermine was better explained by alkalization of the extracellular solution. Additionally, spermine had no effect on the potentiation of GABA-currents by diazepam, indicating that even if diazepam binding is enhanced by spermine, it does not translate to changes in functional activity. Our results clearly demonstrate that while extracellular spermine does not have direct effects on human native synaptic GABA_A_Rs, spermine-mediated shifts of pH inhibit GABA_A_Rs. Potential spermine-mediated increase of pH in synapses in vivo may therefore participate in increased neuronal activity observed during physiological and pathological states, and during metabolic alterations that increase the release of spermine to the extracellular milieu.

## Introduction

Polyamines (putrescine, spermidine, spermine, and agmatine) are positively charged molecules that have fundamental roles in brain homeostasis by modulating neurotransmission, cellular excitability and membrane permeability^1,2^. Due to their role in neuronal signaling, metabolic alterations that affect intracellular or extracellular concentrations of polyamines have been associated to maladaptive stress, mental disorders, and neurodegeneration^3–8^. Clinical evidence and animal models have shown that abnormally increased levels of polyamines could lead to self-sustained stress responses of circuits within frontal cortical-limbic structures^1,9,10^, which is a phenomenon frequently observed in major depressive disorder (MDD) and mood disorders^11,12^. Moreover, abnormal spermine metabolism has been linked to the neurotoxicity observed in Alzheimer’s disease^13,14^. Because sustained polyamine stress responses and neurotoxicity are mediated, at least in part, by the interaction between polyamine levels and membrane receptors involved in the control of cellular excitability, a better understanding of polyamine membrane targets is essential for the development of new pharmacological therapeutic interventions in brain disorders targeting polyamine modulation. Most polyamine targets in cellular membranes are proteins that interact with cations (e.g., ion channels permeable to Na^+^, K^+,^ and Ca^2+^) and modulate neuronal excitatory drive. Indirect evidence also suggests that ion channels with specific permeability to anions, and consequently modulating inhibitory transmission, could also be modulated by polyamines, particularly spermine^15–17^. Thus, direct electrophysiological evidence of spermine on GABA_A_ receptors (GABA_A_Rs) may indicate a novel polyamine mechanism of action and lead to novel therapeutic and translational opportunities if confirmed.

GABA_A_Rs are heteropentameric anionic channels that are essential for inhibitory signaling in the brain^18,19^, and the homeostatic control of synaptic excitatory to inhibitory balance^20^. An initial report showed that ion responses mediated by GABA_A_Rs, heterologously expressed in *Xenopus* oocytes using mRNA from rat brain, were potentiated by spermine^15^. It was later found that spermine modulated the binding of diazepam to GABA_A_Rs in synaptoneurosomal preparations, but the effect was abolished after treatment with non-ionic detergents, even though diazepam binding was still preserved^17^. Later work by Discenna et al., showed that spermine reduced GABA-mediated inhibitory postsynaptic potentials (IPSPs) by 55% in rat hippocampal slices^16^. The authors attributed these changes to inhibitory effects on presynaptic voltage-gated calcium channels (VGCC), which in turn would reduce the release of GABA stored in synaptic vesicles. Nonetheless, the concentration of polyamines needed to reduce half of VGCC ion responses is very high and unlikely to have physiological effects at the synaptic level (spermine, ≈4.7 mM < spermidine, ≈11 mM < putrescine, ≈90 mM for N-type VGCC;^21^). The estimated concentration of spermine in synaptic vesicles is in the 1.5–2.8 mM range^22^, which suggests that additional mechanisms and targets such as extracellular H^+^, or direct modulation of GABA_A_Rs need to be explored. The primary goal of our study was to determine whether extracellular spermine, the polyamine producing the largest effects in previous studies^15–17,21^, exhibits a modulatory effect on native human synaptic GABA_A_Rs. For this, we microtransplanted human receptors from the dorsolateral prefrontal cortex, still embedded in their native membranes and associated with their accessory proteins^23–26^, and studied the effects of extracellular spermine on GABA-elicited ion currents.

## Materials and methods

### Oocyte preparation

Frogs were placed in anesthetic solution (0.17% MS-222) for 10–15 min before extracting the ovaries; following procedures in accordance with the National Institutes of Health Guide for the Care and Use of Laboratory Animals, the IACUC at the University of California, Irvine (IACUC: 1998–1388), and at the University of Texas Medical Branch at Galveston (IACUC:1803024). Oocytes were isolated and defolliculated by carefully stirring them in a solution containing 2 mg/mL collagenase for 2 h at 30 °C. Then, oocytes were transferred to a Petri dish containing Barth’s solution [88 mM NaCl, 1 mM KCl, 0.41 mM CaCl_2_, 0.82 mM MgSO_4_, 2.4 mM NaHCO_3_, 5 mM HEPES (pH 7.4)], and placed in a temperature-controlled environment at 16 °C for 24 h. Stage V–VI oocytes were manually separated and placed in a fresh Barth’s solution for injection of synaptoneurosomal enriched membranes.

### Microtransplantation of synaptic membranes (MSM)

The subjects for this study consisted of postmortem human dorsolateral prefrontal cortex (DLPFC), from four psychiatrically healthy subjects (Table 1). The brains and samples were collected by the University of California, Irvine Brain Bank (UCIBB) in accordance with the University’s Institutional Review Board after obtaining consent from next of kin. These brains have been characterized using UCIBB psychological autopsy protocol that has been extensively used by our group in past years^27–30^. Human synaptoneurosomes, harboring GABA receptors, were isolated from ≈50 mg frozen DLPFC from each brain donor using Syn-PER method (Thermo Fisher Scientific). The resultant pellet, enriched in synaptoneurosomes, was suspended in sterile distilled water and sonicated to create small proteoliposomes that can fuse to the oocytes’ extracellular membrane. After the protein concentration was determined by using Qubit protein assay reagent kit (Thermo Fisher Scientific) the membrane preparations were stored at −80 °C until the moment of injection. One day before electrophysiological recordings the synaptic membranes were injected into stage V–VI *Xenopus laevis* oocytes using protocols previously published for cellular membranes^23,31,32^. Each oocyte was injected with 50 nL of synaptic proteoliposomes (2 mg/mL protein concentration) and characterized 18–36 h post-injection.

**Table 1:** Demographics

Donor	Age/Gender	PMI (hours)	pH	RIN
S1	50/M	29	6.6	8.3
S2	52/M	18.8	6.53	9.2
S3	64/M	10.5	7.13	9.8
S4	56/M	9	6.64	9.7

*S* subject, *M* male, age is counted in years and postmortem interval (PMI) in hours. *RIN* RNA integrity number

### Heterologous expression of GluR3

To test and monitor the biological activity of extracellular spermine we expressed GluR3 receptors in oocytes as previously reported^33^. Briefly, 50 nL of cRNA (1 mg/ml) for human GRIA3 (Clontech, Mountain View, CA) were injected into the equatorial band of defolliculated *Xenopus* oocytes and Kept in Barth’s solution until the moment of recording 2–4 days after injection.

### Electrophysiological recordings

Agonist-elicited ion currents were recorded by the two-electrode voltage clamp (TEVC) method^34^. Microelectrodes were filled with 3 M KCl and resistance of the microelectrodes ranged from 0.5 to 3.0 MΩ. Piercing and recording took place in a chamber (volume ≈0.1 ml) continuously perfused (5–10 ml/min) with Ringer’s solution [115 mM NaCl, 2 mM KCl, 1.8 mM CaCl_2_, 5 mM Hepes (pH 7.4)] at room temperature (19–21 °C). Oocytes were voltage clamped to −80 mV. Ion currents were recorded and stored with WinEDR version 2.3.8 Strathclyde Electrophysiology Software (John Dempster, Glasgow, United Kingdom). Most drugs were from Sigma (St. Louis. MO). Kainic acid, baclofen and CGP-55845 hydrochloride were from Tocris (Minneapolis. MN). Working solutions were made by diluting aqueous 1 M spermine or ethanolic 10 mM diazepam stocks in Ringer’s solution. Same results were obtained when using spermine, freshly prepared, or from frozen aliquots, from three different lots. The biological activity of spermine was tested by its antagonist effect on GluR3 receptors which, similarly to all Ca^2+^-permeable AMPA receptors, are sensitive to extracellular polyamines^35,36^. After addition of spermine the Ringer’s solution pH was fixed at pH 7.4 by the addition of hydrochloric acid^21^. In some experiments the pH of the extracellular solution was adjusted to 10 or 6 by adding either sodium hydroxide or hydrochloric acid and used immediately.

### Data analysis

The EC_50_, EC_25_ and the Hill coefficient were determined by fitting the Hill equation in the form I = Imax/(1 + (EC_50_/[A])^n^), in which I is the current amplitude, Imax is the maximum current amplitude at the concentration of the agonist [A], EC_50_ is the agonist concentration that induces 50% of the maximal response, and n is the Hill coefficient. The EC_50_ and EC_25_ were estimated for each brain donor (biological replicates). The number of microtransplanted oocytes tested (technical replicates) was determined by analyzing the magnitude of the effect and the dispersion of the variability, and using paired data when possible, similarly to pharmacological analysis of heterologous expression of GABA and AMPA receptors in *Xenopus* oocytes^37,38^. The experimental data are shown as the mean ± S.E.M. Statistical differences were determined by two-sided Student *t*-test and considered significant when *p* < 0.05 (JMP version 14; SAS Institute, Cary, NC).

## Results

### Effects of spermine and pH on GABA currents

**Fig. 1:**
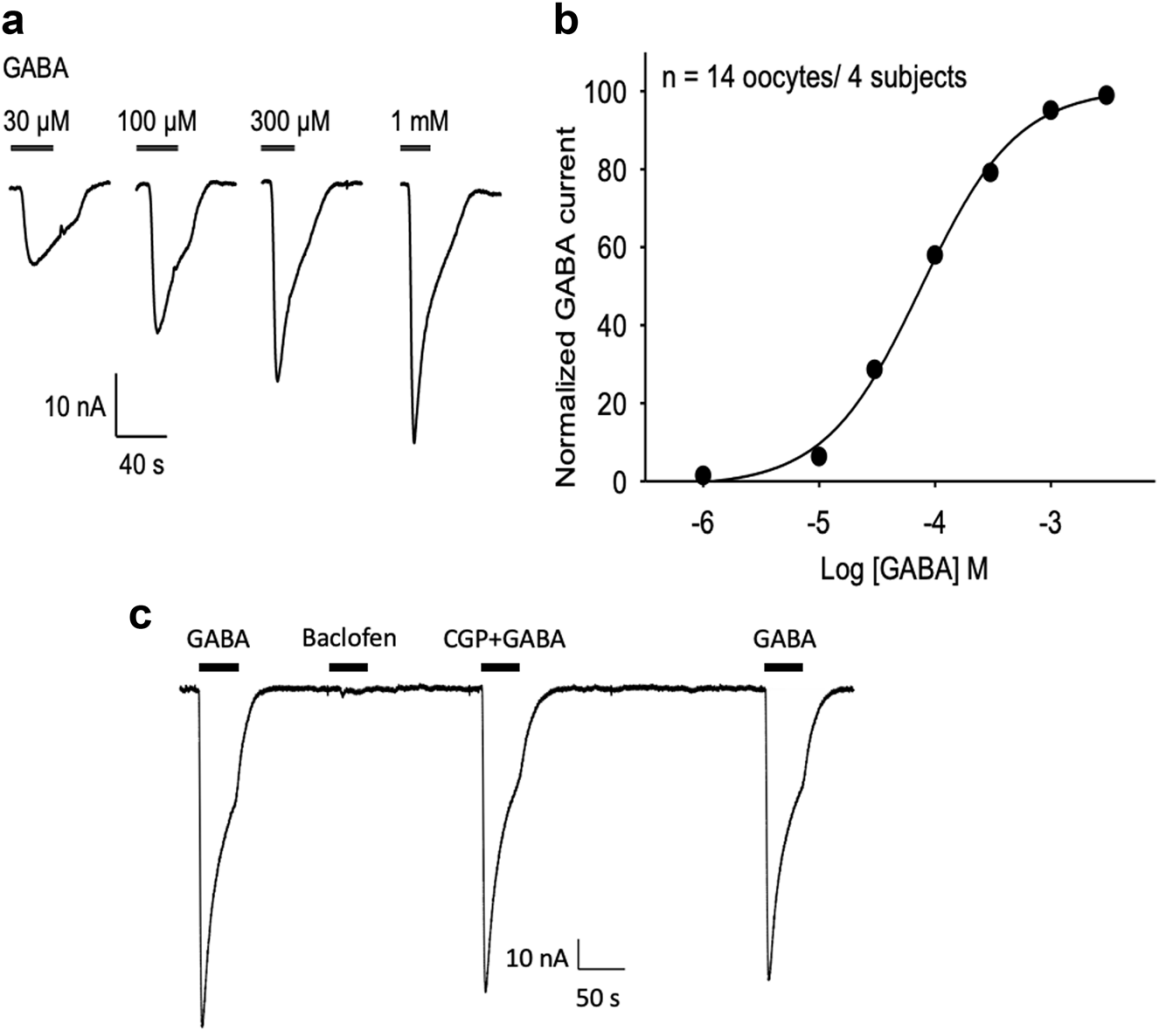
Reactivation of GABA_A_ receptors from frozen human synaptoneurosomes from the DLPFC. **a** Synaptoneurosomal preparations, isolated from the dorsolateral prefrontal cortex of four subjects with no history of psychiatric disorders, were microtransplanted into *Xenopus* oocytes. Native GABA receptors were activated by perfusing sequentially increasing concentrations of GABA. **b** Average concentration response curve for GABA, using data from 4 subjects, 3–4 oocytes each. Mean ± s.e.m., error bars are smaller than the symbols. The average EC_50_ for all subjects pooled was 77.9 ± 4.7 µM, n_H_ = 1 ± 0.03 (mean ± s.e.m.). The individual EC_50_ for each subject was: S1 = 68.3 ± 4 µM, n_H_ = 1 ± 0.02 (*n* = 4 oocytes); S2 = 80.1 ± 9 µM, n_H_ = 1 ± 0.01 (*n* = 3 oocytes); S3 = 96.7 ± 3 µM, n_H_ = 1.1 ± 0.05 (*n* = 4 oocytes) and S4 = 63 ± 11 µM, n_H_ = 1.2 ± 0.1 (*n* = 3 oocytes). **c** Baclofen (100 μM), an agonist of metabotropic GABA_B_ receptors elicit negligible responses in microtransplanted oocytes. CGP-55845 (5 μM) did not significantly affect GABA elicited currents (97.3 ± 2.1% of control; *n* = 16 oocytes/4 subjects) in microtransplanted oocytes

**Fig. 2:**
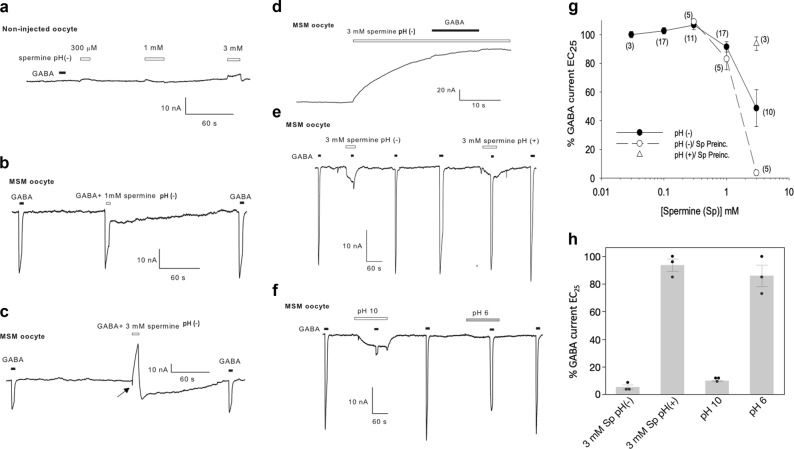
Spermine and pH effects on native GABA currents. **a** Non-transplanted oocytes were unresponsive to extracellular perfusion of GABA (30 μM) but elicited concentration-dependent biphasic responses to perfusion of spermine when the pH of the Ringer’s solution was not readjusted to 7.4 after addition of spermine (pH (−)). **b**, **c**. Oocyte microtransplanted with synaptic membranes (MSM) from subject S3 showing responses to 30 μM GABA (EC_25_ for S3) and to the co-application of 30 μM GABA with different concentrations of spermine pH(−). Notice that an initial inward current (arrow in C), elicited during co-application of GABA and 3 mM spermine pH(−), was cut short by the appearance of a large outward current. **d** Preincubation with 3 mM spermine pH(−) inhibited the responses to 30 μM GABA. **e** GABA responses (30 μM) in microtransplanted oocytes were inhibited during perfusion of 3 mM spermine pH(−). However, when the pH was titrated to 7.4 (pH( + )) GABA responses were minimally reduced. **f** Perfusion of extracellular solution, titrated to pH 10, and to the same oocyte shown in E, elicited a slow inward current and reduced GABA responses to 9.9 ± 0.4% of the control. **g** Plot summarizing the effects of spermine on GABA’s EC_25_. Circles indicate the mean ± s.e.m., of GABA currents derived from all subjects when the pH of the Ringer’s solution was not readjusted after addition of spermine (pH (−)). Spermine at concentrations of 100 µM, 1 mM, and 3 mM, changed the pH to 7.56, 8.9, and 10.3, respectively. Black circles represent the effects produced by co-application of spermine and GABA, and white circles the added effects of the preincubation with spermine for at least 10 s, or when the baseline current was stable. The white triangle indicates the effects of 3 mM spermine, preincubated and then co-applied with GABA, in Ringer’s solution with the pH fixed at 7.4 (pH ( + )). The number in parenthesis indicates the number of oocytes tested. **h** Comparison of the effects produced by high concentration of spermine with and without readjusting the pH, and with solutions without spermine but high pH in subject S3 which had the strongest effect to 3 mM spermine pH(−)

Perfusion of 1 mM GABA to oocytes microtransplanted with human postmortem synaptoneurosomal membranes preparation elicited fast activating ion currents (GABA currents) with a maximum amplitude of 100 nA ± 14 nA (*n* = 14 oocytes/4 subjects). The amplitude of GABA currents was dependent on the GABA concentration with an EC_50_ of 78 µM (Fig. 1a, b). In contrast, non-injected oocytes showed no responses to GABA (Fig. 2a), confirming that GABA currents in microtransplanted oocytes were exclusively mediated by human transplanted receptors. To determine the potential contribution of GABA-mediated metabotropic responses present in synaptoneurosomes we used 100 μM baclofen, a specific agonist, and 5 μM CGP-55845, a specific antagonist, for GABA_B_ receptors (Fig. 1c). Baclofen elicited negligible responses in microtransplanted oocytes and CGP-55845 did not affect GABA-elicited currents (97.3 ± 2.1% of control; *n* = 16 oocytes/4 subjects), indicating that microtransplanted GABA_B_ receptors may be uncoupled to the oocytes’s intracellular signaling, and GABA elicited currents in these oocytes were due to direct activation of GABA_A_Rs.

**Fig. 3:**
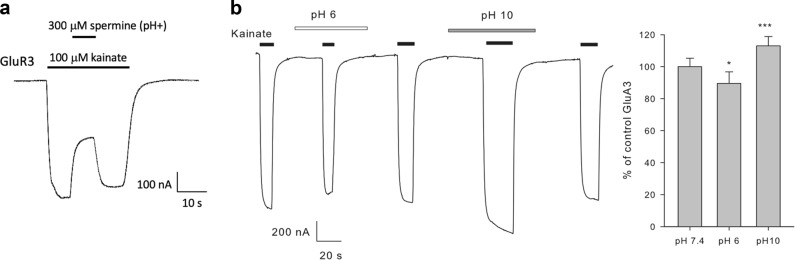
Effects of spermine and pH on homomeric GluR3. **a** Ion currents elicited with 100 mM kainate were inhibited by spermine with pH adjusted to 7.36 by 51 ± 2.5% (*n* = 6). **b** Perfusion of extracellular solution with pH adjusted to 6 inhibited kainate currents to 89 ± 7.2% of the control and pH adjusted to 10 increased it to 114 ± 5.8 % (*n* = 5; *p* < 0.01 paired, two-sided *t* Student’s *t*est)

Because the directionality of a putative modulation by polyamines was not known we tested spermine on the EC_25_ for GABA in each subject (≈30 μM), a concentration that allows the characterization of positive or negative modulation of native GABA receptors. In our first experiments, we initially tested the effects of extracellular spermine without readjusting the pH after spermine addition (pH (−)) (Fig. 2a–d). In the pH (−) condition, the perfusion of spermine by itself at concentrations of 100 µM (pH = 7.56), or lower, did not have effects on transplanted oocytes. Higher concentrations of spermine elicited non-specific slow activating currents in transplanted and non-transplanted oocytes. The non-specific effects were similar to results reported previously with spermine alone^15^, or alkaline pH^39^, eliciting inward or biphasic currents^15^, or in some batches of oocytes that were highly sensitive to alkaline pH, spermine elicited strong outward currents^39^ (Fig. 2c, d). Whereas co-perfusion of 100 µM spermine in pH (−) solution had only minimal effects on GABA currents (102 ± 2.2% of control; *n* = 11 oocytes/4 subjects), 1 mM and 3 mM spermine reduced GABA currents to 91 ± 4% and 49 ± 13% of the response elicited by GABA alone (*n* = 17 and 10 oocytes/4 subjects). Preincubation with 3 mM spermine pH (−) for at least 10 s, to wait for the stabilization of the non-specific current, blocked GABA elicited currents to 4 ± 2% of the control (*n* = 5 oocytes/the subject with the strongest spermine pH(−) effect) (Fig. 2d, e, g), initially suggesting that the application of spermine at high concentrations had a direct effect on GABA currents. However, further experiments testing pH and spermine in the same oocytes showed that spermine-induced alkalization of Ringer’s pH was responsible for the non-specific current and the negative modulation of GABA responses (Fig. 2f–h). Titration of Ringer’s solution to 7.4 after spermine addition prevented the negative modulation of GABA receptors even at 3 mM spermine, eliciting GABA currents 94 ± 5% of the control (*n* = 3 oocytes from the subject with the strongest spermine pH(−) effect; S3). In contrast, Ringer’s solution with alkaline pH (pH = 10), which is similar to the change in pH produced by 3 mM spermine, blocked GABA currents to 9.9 ± 0.4% of the control (*n* = 3 oocytes/subject S3). The blockade by 3 mM spermine pH(−) was not statistically different from the blockade elicited by pH 10 alone (5.3 ± 1.6% *vs* 9.9 ± 0.4 %; *p* = 0.139, paired, two-sided Student’s t-test). To confirm that spermine was biologically active at pH 7.4 we tested its effects on heterologously expressed glutamate receptors (GluR3) (Fig. 3); spermine (300 μM) blocked kainate-elicited currents by 51 ± 2.5% (*n* = 6; mean ± s.e.m.). Interestingly, acidification of the pH reduced the kainate response to 89 ± 7.2% of the control and alkalization increased it to 114 ± 5.8% (*n* = 5; *p* < 0.01 paired, two-sided *t* Student’s test). These results indicate that extracellular spermine is biologically active and, by itself, does not directly modify the amplitude of GABA currents. It is the increase in the pH of the buffered solutions that negatively modulates GABA_A_Rs.

### Effects of spermine on the modulation by Diazepam

**Fig. 4:**
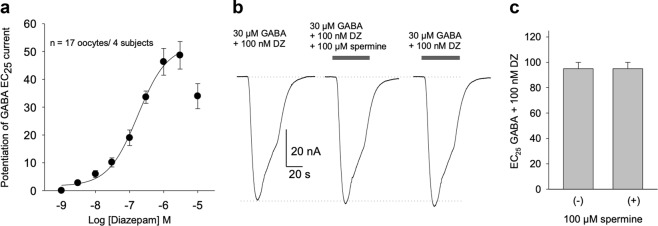
Spermine has no effect on diazepam potentiation of GABA_A_Rs. **a** Average concentration-response curve of diazepam potentiation of GABA currents, using data from 4 subjects, 4–5 oocytes each. Mean ± s.e.m. The current elicited by GABA’s EC_25_ in each oocyte was defined as zero in the plot. The average EC_50_ for all subjects was 275 ± 66 nM (mean ± s.e.m; *n* = 17). The individual EC_50_ for each subject was: S1 = 253 ± 11 nM (*n* = 4), S2 = 273 ± 16 nM (*n* = 4), S3 = 159 ± 55 nM (*n* = 4) and S4 = 670 ± 121 nM (*n* = 5). Higher concentrations of diazepam reduced the efficacy of the potentiation. **b** Representative traces of currents elicited by GABA’s EC_25_ co-applied with 100 nM diazepam before and in the presence of 100 µM spermine. **c** The plot shows no change of the normalized maximal current responses elicited by GABA’s EC_25_ plus 100 nM diazepam and 100 µM spermine (+) compared to those without it (−) (*n* = 6 oocytes/3 subjects)

Diazepam positively modulated GABA responses by 49 ± 5% of the control, with an average EC_50_ of 275 nM (Fig. 4). High concentrations of diazepam (>3μM) reduced the level of potentiation, as has been previously observed for heterologously expressed GABA_A_ receptors^40^. Because in previous studies 100 µM spermine was the concentration that elicited the largest increase of diazepam binding on GABA^17^, we used the same concentration to maximize its putative effects of current potentiation. Spermine (pH 7.4) had no effects on the amplitude of GABA currents in presence of 1 µM diazepam (change of current to 107 ± 9.2% of the control, *n* = 27 oocytes/4 subjects). To avoid the possibility that desensitization of GABA currents could counteract spermine effects, we tested the effect of spermine with a low concentration of diazepam (100 nM) in order to see if the binding for diazepam was enhanced by spermine then the GABA current amplitude should be potentiated proportionally. In these conditions, we did not see spermine effects on the amplitude of GABA currents (*n* = 6 oocytes/3 subjects). To discard the possibility that spermine did not potentiate GABA currents because the potentiation was already at its maximum level, further potentiation by 1 µM diazepam after spermine was confirmed (*n* = 6 oocytes/3 subjects).

## Discussion

Polyamines, by modifying the current properties of ionic receptors, play important neuromodulatory roles in health and disease^1^. Spermine modulation of synaptic GABA_A_Rs could have significant consequences on inhibitory neurotransmission and important translational relevance for neuropsychiatric and neurodegenerative disorders. Our results, however, clearly show that spermine has no direct modulatory role on the functional responses of synaptic human GABA_A_Rs. Instead, it is the alkalization of the extracellular solution by spermine that can explain some effects previously reported. Spermine effects on GABA_A_Rs in *Xenopus* oocytes was first observed simultaneously with the activation of a non-specific biphasic current^15^. We were able to replicate the non-specific current seen in Brackley et al., in our own experiments when the pH was not corrected after spermine addition. This effect was similar to the cAMP-mediated K^+^ outward current elicited by alkaline extracellular solutions and was more evident in oocytes highly sensitive to alkaline treatment^39^. We also did not find evidence that spermine modifies the amplitude of GABA currents in presence of diazepam, indicating that even if diazepam binding is enhanced by spermine, it does not translate to changes of functional activity. Most cortical synaptic GABA_A_R subunits are arranged in α1γ2β2α1β2 counterclockwise manner, as seen from the outside of the cell^41,42^. Accordingly, we have confirmed, by proteomics, the presence of α1, β2, β3 and γ2 subunits in our synaptic preparations^43^. Diazepam can bind these GABA_A_Rs receptors at a high-affinity site at the α1/γ2 interface^42^, or at transmembrane low-affinity sites at the other subunits interfaces^44,45^. Because it was previously reported that polyamine-mediated modulation by diazepam binding disappeared after treatment with non-ionic detergents it is possible that lipids were mediating spermine effects^17^. The activity and pharmacology of GABA_A_Rs is affected by the composition and dynamics of surrounding lipids^46,47^, and intracellular spermine stabilizes the cellular membrane fluidity^48^; therefore an interaction between increased intracellular spermine and lipids in rat synaptoneurosomal preparations could have mediated the effect on diazepam-binding experiments.

Alkaline pH can also affect benzodiazepine effects, at pH 8.4 flunitrazepam had a slightly higher potentiation of GABA currents in cerebellar granule cells (17%), than at pH 7.4^49^, suggesting more binding of flunitrazepam at higher pH. Besides the pH effect, discrepancies between our results and previous reports could also be due to potential interspecies differences between GABA_A_Rs in humans and animal models used in previous studies^50,51^ (e.g., species-specific posttranslational modifications or interactions with their accessory proteins^52,53^). The potential effect of spermine on GABA_A_Rs has important clinical implications especially in the context of complex neurological and mental disorders with known alterations in GABAergic neurotransmission. Therefore, testing spermine on human receptors with their own posttranslational modifications, accessory proteins, and surrounding human lipids, is a needed step to avoid confounding factors and better dissect the role of polyamines in disorders like suicide and depression^1,30^. This type of in vitro pharmacological profiling using post-mortem native human receptors has important applications to study normal and diseased conditions but also to characterize, in vitro, new drugs for the pharmacological treatment of neuropsychiatric and neurodegenerative conditions. Because our experiments indicate that spermine does not have a direct functional effect on human GABA_A_Rs, the rule of thumb that spermine only affects cationic channels still holds up. It is important to note that our results only apply to cortical human synaptic receptors composed by α1, β2, β3, and γ2 subunits which: (1) are translationally relevant due to their putative modulation by spermine, (2) are the most abundant in the human cortex^50^, and (3) we have confirmed to be present in our synaptic preparations^43^. While there is evidence of presynaptic receptors in the spinal cord, hippocampus, cerebellum and layer 4 of the primary visual in cortex^54–56^, to our knowledge, there is no direct evidence for the presence of presynaptic GABA_A_Rs in the frontal, temporal or parietal cortices; therefore, it is highly likely that the majority of microtransplanted receptors are postsynaptic. Our results cannot discard the possibility that spermine might have effects on extrasynaptic receptors, or in any other of the number of isoforms composed by the combination of the 19 genes for GABA_A_Rs.

Interestingly, high alkaline pH blocked GABA currents in microtransplanted oocytes, functionally confirming that GABA_A_Rs in our synaptoneurosomal preparations are predominantly composed by α1β2γ2 subunits^43,57^. Although the physiological relevance of GABAergic inhibition by alkaline pH is not completely understood^58^, it is known that during synaptic transmission a fast and strong acidosis of the synaptic cleft is followed by a long but transient rise of pH^59^ which likely results from membrane transport and fluxes of H^+^ and bicarbonate^58^. Moreover, rises of pH increase neuronal activity and excitability^58^. Because spermine can be accumulated in synaptic vesicles and be released during depolarization^22^, it would not be surprising that synaptic release of spermine participates in the transient rise of pH to remove H^+^-induced inhibition of NMDA receptors^60^ and inhibit GABA_A_Rs to synergistically increase neuronal activity in normal physiological conditions.

In conclusion, our experiments demonstrate that spermine has no direct effect on human native synaptic GABA_A_Rs. However, spermine-mediated shifts of pH inhibit GABA_A_Rs. If a similar rise in pH is also observed in vivo in the synaptic cleft micro-environment it could participate in the increased neuronal activity observed during alkaline physiological and pathological states, and during metabolic alterations that increase the release of spermine to the extracellular milieu.
